# Linking psychological need experiences to daily and recurring dreams

**DOI:** 10.1007/s11031-017-9656-0

**Published:** 2017-11-30

**Authors:** Netta Weinstein, Rachel Campbell, Maarten Vansteenkiste

**Affiliations:** 1 School of Psychology, Cardiff University, 70 Park Place, Cardiff, CF10 3AT Wales, UK; 2 Ghent University, Ghent, Belgium

**Keywords:** Self-determination theory, Need satisfaction, Need frustration, Dreams, Emotions

## Abstract

The satisfaction of individuals’ psychological needs for autonomy, competence, and relatedness, as conceived from a self-determination theory perspective, is said to be conducive to personal growth and well-being. What has been unexamined is whether psychological need-based experiences, either their satisfaction or frustration, manifests in people’s self-reported dream themes as well as their emotional interpretation of their dreams. A cross-sectional study (*N* = 200; *M* age = 21.09) focusing on individuals’ recurrent dreams and a three-day diary study (*N* = 110; *M* age = 25.09) focusing on daily dreams indicated that individuals experiencing psychological need frustration, either more enduringly or on a day-to-day basis, reported more negative dream themes and interpreted their dreams more negatively. The contribution of psychological need satisfaction was more modest, although it related to more positive interpretation of dreams. The discussion focuses on the role of dreams in the processing and integration of psychological need-frustrating experiences.

## Introduction

Dreams may help to process and integrate people’s daily experiences (e.g., De Monchaux 1993; Wamsley and Stickgold 2011), and it is thought that meaningful experiences, particularly those that are threatening (Revonsuo 2000), are at the forefront of dream material (Erikson 1954; Jung 1948/1974). Research based in self-determination theory (SDT; Ryan and Deci 2000, 2017) has focused on day-to-day and enduring experiences of three basic psychological needs for autonomy, competence, and relatedness, which are thought to be especially important for well-being and psychological growth (Deci and Ryan 2000, 2012; Sheldon et al. 1996). More recently, these psychological need experiences have been further defined in terms of being actively satisfied or frustrated (Bartholomew et al. 2011; Vansteenkiste and Ryan 2013).

Herein, we test the idea that such experiences might play out in dreams, with especially psychological need frustrating events surfacing in the frightening, sad, or anger-promoting dream themes individuals report (Belicki et al. 1985; Brimacombe 1993; Hartmann 1984; Levin and Hurvich 1995). Dating back to the traditions of Freud (1913/2010), Jung (1948/1974), and Perls et al. (1973), these dreams reflect experiences that were difficult to integrate or process in waking time because they were particularly painful or threatening. That is, such bad dreams represent “left-overs” from poorly or even unprocessed daily experiences. There is less, but some research suggesting positive events are evident in dreams because these, as well, are processed more deeply in sleep (Curci and Rimé 2008; Zadra and Donderi 1996).

To explore whether experiences of related to psychological needs in waking life are related to the deeper level of processing that dreams provide, in this contribution participants were asked to recall and describe their recurring dreams (Study 1 involving a cross-sectional design) and their dreams from the night before (Study 2 involving a diary design) to examine whether these waking experiences would play out in dreams. Overall, we expected that psychological need frustrating experiences in waking life would be represented in dynamically analogous dream contents; that is, they would relate to more negative psychological need-relevant themes and more negative emotions in dreams. In contrast, we thought psychological need satisfaction in waking life may be linked to more positive emotions in dreams given that positive experiences might also be processed through dreaming, though this hypothesis was more tentative.

## Basic psychological needs

Theorizing and research in SDT (Deci and Ryan 2000; Ryan and Deci 2000) gives reason to believe that three psychological needs experienced during waking life—that is, those for autonomy, competence, and relatedness—may make for especially meaningful events to be expressed in dreams. All three of these experiences reflect important occurrences in people’s daily functioning (Reis et al. 2000): The psychological need for *competence* refers to feeling effective in acting on the world and achieving desired outcomes. The psychological need for *autonomy* refers to experiencing a sense of choice and psychological freedom in one’s functioning. Finally, the psychological need for *relatedness* refers to feeling close and connected to others in one’s social sphere. Dozens of studies, using cross-sectional (e.g., Milyavskaya et al. 2013), diary (e.g., Van der Kaap-Deeder et al. 2017), and experimental methods (e.g., Weinstein et al. 2010) have shown psychological need satisfaction to promote human flourishing, as indexed by higher levels of vitality, life satisfaction, and engagement. The benefits of psychological need satisfaction were found to hold across cultures (e.g., Chen et al. 2015; Deci et al. 2001; Jang et al. 2009) and different age groups (e.g., Véronneau et al. 2005).

Historically, SDT scholars have primarily focused on the experience of psychological need satisfaction. Yet, it has become increasingly clear that individuals’ experience of psychological need frustration deserves to be studied in its own right for two conceptual reasons (Bartholomew et al. 2011; Vansteenkiste and Ryan 2013). First, the absence of psychological need satisfaction does not necessarily entail the presence of psychological need frustration. For psychological need frustration to occur basic needs have to be actively undermined rather than merely deprived. For instance, although people may not feel particularly choiceful in carrying out an activity, it does not necessarily mean that they are engaged in the activity against their own will. Consistent with this claim, studies show that experiences of psychological need satisfaction and psychological need frustration are moderately (rather than perfectly) negatively related (Bartholomew et al. 2011; Gillet et al. 2012; Haerens et al. 2015). Second, increasing evidence has been garnered for a dual process model, with psychological need frustration yielding harmful effects in the prediction of maladaptive outcomes above and beyond the contribution of psychological need fulfillment, and psychological need satisfaction especially relating to adaptive outcomes. To illustrate, psychological need frustration yields a unique relation with depressive symptoms (Bartholomew et al. 2011), disordered eating (Boone et al. 2014), and ill-being (Stebbings et al. 2015), a finding that emerged in cross-sectional, longitudinal, and diary studies (Verstuyf et al. 2013).

## Waking psychological need experiences and dreaming

Building on previous research, we explore the idea that the psychological impact of experiencing psychological need frustration is sufficiently meaningful and threatening that it may be further processed through dreaming. Though dreams may be reflective of these daily need-frustrating experiences to some extent (Revonsuo 2000), the events, objects, and characters apparent in dreams may take different forms than the daily experiences they represent. Freud (1913/2010) was first to describe this distinction between the actual events and objects of the dream and the underlying meaning of those themes. According to Freud and other psychodynamic theorists, *dream themes* offer some disguise for the real-life events because these are otherwise painful or unacceptable to the dreamer (Allport 1937). Despite the potential ambiguity of dream themes, it is apparent that certain events or objects are common to dreams; these have been identified in large-scale surveys (Nielsen et al. 2003), and through therapists’ collections (Spangler et al. 2009) and include falling, fire, swimming, being chased or pursued, being nude or inappropriately dressed, and being frozen with fright, among other themes. Indeed, when testing students, themes such as “being attacked”, “falling”, or failure-related themes involving school are commonly reported by approximately three to four out of five respondents (Griffith et al. 1958; Nielsen et al. 2003). Such themes may be thought to reflect feelings of helplessness, rejection from others, or inefficacy, and have also been shown to emerge in clinical populations (Blackburn and Eunson 1989).

Here, we argue that these themes may also represent dynamics which are especially relevant to the frustration of the psychological needs of autonomy, relatedness, and competence. Although this assertion has not yet been tested, a previous study (Kasser and Kasser 2001) linking more stable individual differences to dream themes may inform the present work. In this study, researchers tested the dream themes of materialistic individuals and found that those who find the pursuit of wealth to be especially important, and who, in previous research, have been shown to experience more psychological need frustration (Unanue et al. 2014), report more negative dream themes (e.g., falling), whereas those low in materialism reported more positive themes (Kasser and Kasser 2001).

Along with the themes in dreams being less or more negative, people’s *perceptions* or *interpretation* of their dreams along these dimensions may be meaningful reflections of their important daily experiences. In fact, some argue that even if a dream event does not accurately reflect the dynamics of waking experiences, understanding individuals’ projections of their psychological states onto these dream themes may offer important insight into their waking experiences (Perls et al. 1973; Simkin 1972). In this view, dreams provide a personalized and dynamically rich, but often ambiguous, set of objects and events on which individuals can project their experiences.

In line with the assertions discussed above, and possibly influenced by both dream themes and subjective interpretations, previous work shows a relation between individual differences in positive and negative emotions, including anxiety or stress, and positive and negative evaluations of dreams (Armitage et al. 1995; Brown and Donderi 1986; Dunn and Barrett 1988; Kallmeyer and Chang 1998; Levin and Hurvich 1995; Zadra and Donderi 2000). Moreover, experimental manipulations of pre-sleep emotions have been shown to predict negative dream recall (Baekeland 1971; Carpenter 1988). Finally, individual differences in mindfulness (Brown and Ryan 2003) relate to both lower anxiety in dreams (Simor et al. 2011) and more psychological need satisfaction (Campbell et al. 2015, 2017), which provides additional basis for our expectation that psychological need experiences would be related to dreams.

## Present studies

The research reviewed above indicates that waking experiences may be reflected in dream themes and perceptions of their emotional tone. Importantly, while dream themes and emotions may relate (for example, a dream in which one is falling is likely to be more negatively valenced), dream themes and dreamers’ emotions are conceptually (Perls et al. 1973) and empirically (Schredl et al. 2009, 1999) distinct from one another. That is, not all negative dream themes necessarily come with negative emotions, while not all positive dream themes are necessarily interpreted as positive by all individuals.

In the present studies, we examined the links between the frustration and satisfaction of the basic psychological needs for autonomy, competence, and relatedness, and both dream themes and emotions. In Study 1, using a cross-sectional design, we aimed to examine the link between general psychological need satisfaction and frustration and important recurring dreams, as a way of testing how these enduring experiences are reflected in dreams. Given that considerable variation exists in individuals’ daily psychological need satisfaction and, presumably, also in their daily dreaming, Study 2 used a diary design to examine whether need-based experiences in the day would manifest during the subsequent night’s dreams. In both studies, we tested the hypotheses that (1) psychological need frustration would link to negative dream themes and emotions, and that (2) psychological need satisfaction would be positively linked to positive dream emotions. The relation between psychological need satisfaction and negative dream themes is more tentative given that past research has provided evidence for a dual pathway, with psychological need frustration primarily predicting maladaptive outcomes and psychological need satisfaction primarily relating to adaptive outcomes (e.g., Bartholomew et al. 2011; Boone et al. 2014; Jang et al. 2016; Stebbings et al. 2015).

## Study 1

### Method

#### Participants and procedure

Two hundred students (131 women) aged 18 to 33 years (*M* = 21.09, *SD* = 2.46) participated in this study, and responded to a short survey assessing their general psychological need satisfaction and psychological need frustration and rated items regarding a common recurring dream. All participants were asked to “Think to your most common recurring dream… a dream that you have had again and again in your past. Write as much as you can about the things that happened in your most memorable dream. Please put in as much detail as you can remember! Tell us the entire story of your dream…” Participants wrote about dreams that occurred between 0 and 192 months before the study (*Median* = 2.00; 80.5% of dreams reported were within the last 12 months). Participants then reported on the presence or absence of themes in their dreams (described below) and their positive and negative evaluations of dreams.

### Materials

#### General psychological need satisfaction and frustration

Participants reported on their general psychological need satisfaction and frustration using a shortened version of the basic psychological need satisfaction and need frustration scale (BPNSNFS; Chen et al. 2015). Chen et al. (2015) provided extensive information regarding the 24-item version in four different cultures (i.e., US, Belgium, Peru, and China). Similar to previous work (Van der Kaap-Deeder et al. 2017), a shortened 12-item version involving a balanced combination of frustration and satisfaction items of all three needs (i.e., two items for each valence—frustration vs satisfaction—for each of the three needs) was used in the current study. Congruent with the dual pathway model, Van der Kaap-Deeder et al. (2017) reported that daily psychological need satisfaction and daily frustration were primarily predictive of, respectively, daily well-being and ill-being. Items were rated on a scale ranging from 1 (*not at all true*) to 5 (*extremely true*). Items included: “I feel that my decisions reflect what I really want” (autonomy satisfaction) and “I feel that people who are important to me are cold and distant towards me” (relatedness frustration). Participants responded to how much each was true of them in general, without specification of a specific time-line. Although the needs represent three distinct constructs, experiencing one of them often co-occurs with experiencing another (Baard et al. 2004; Ryan and Deci 2000). For this reason, researchers often combine the needs to create a single composite score (e.g., Deci et al. 2001; Linley et al. 2010). In line with this approach, six items were averaged to create a single psychological need frustration score and six others to create a psychological need satisfaction score. Reliabilities were acceptable for frustration, α = .75 and satisfaction, α = .76.

#### Common negative themes in dreams

**Fig. 1:**
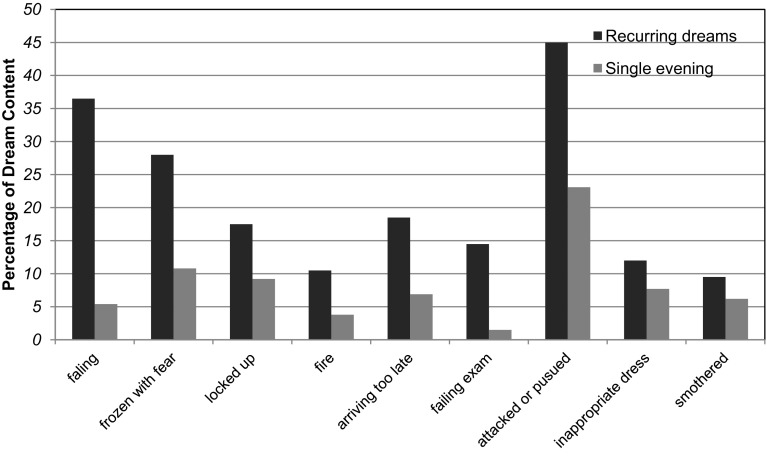
Frequency of negative dream themes for both recurring (Study 1) and single evening (Study 2) dreams. *Note* Data are expressed in percentages of dreams that included this event. Please note that a single dream may include more than one of these contents

Participants reported whether they had experienced any of nine negatively valenced common dream themes (represented in > 10% of common dreams) taken from Domhoff (1996, 1999; see also Hartmann et al. 2001; Miller 1969) and could report on more than one theme in the same dream. These included: falling, being attacked or pursued, being frozen with fear, being locked up, the presence of fire, being nude in public, trying repeatedly to do something, failing an examination, being inappropriately dressed, and arriving too late. *Negative themes* each received a score of “1” if participants in the full study reported having had such content in their dream, or “0” if they had not (Fig. 1).

#### Dream emotions

Reflecting on their dream more broadly, participants reported on five items reflecting how *positive* the dream was, and seven items reflecting how *negative* their dream was, using items adapted from Smith and Ellsworth (1985) for assessing dream-relevant emotional experiences. Positive items included: “exciting”, “hopeful” and “good”. Negative items included: “full of stress”, “sad”, and “frustrating”. Participants appraised “how much was this dream” each of these emotions, with a scale from 1 (*not at all*) to 5 (*very much*). Reliabilities were high, αs = .89 and .90 for, respectively, positive and negative emotions.

### Results

#### Statistical controls

Preliminary correlations (Table 1) showed relations between both gender and the time since the reported dream last occurred with study outcomes (i.e., latency). Female gender linked to more negative dream emotions, a finding broadly in line with previous research showing gender differences, though earlier findings identified a link with dream themes (e.g., Schredl and Piel 2005). Longer latency (dreams that were further in the past) related to less positive emotions and more negative themes, suggesting memories of dream themes differ from waking recollections, which tend to be more *positive* as a function of how far back they are in time (Walker et al. 2003).

**Table 1:** Descriptive statistics and correlations (Study 1)

	Mean (SD)	1	2	3	4	5	6	7
1. Gender								
2. Latency	2.00	− .08						
3. Need satisfaction^a^	0.51 (0.14)	.00	.00					
4. Need frustration^a^	0.33 (0.16)	.08	.02	− .05				
5. Positive emotions^b^	2.32 (1.10)	− .07	− .21**	.14	− .05			
6. Negative emotions^b^	2.84 (1.06)	.15*	.05	− .02	.32**	− .55**		
7. Dream themes	0.72 (0.45)	− .05	.10	− .10	.16*	− .33**	.34**	

^a^Means and standard deviations reflect log-transformed predictor variables

^b^Emotions here refers to emotional experiences within dreams

*p < .05, **p < .01. Latency (variable 2) figures are for the median of the variable rather than the mean

#### Dream themes

Negative themes in dreams were evaluated using logistic regression analysis given they had an absent (0) or present (1) distribution. This was predicted by gender and time since dream last occurred (dream latency) as controls, and psychological need satisfaction and frustration. All predictors were included simultaneously in the same step. Logistic regression analysis assumes predictors have little or no collinearity between them and indeed in this case all correlations between predictors were non-significant, with the strongest being the relation between psychological need satisfaction and frustration (*r* = − .05, *p* = .46). Further, there were very little missing data (one participant had incomplete data; this person was retained in the analysis). Although regression models do not assume independent variables need to be normal, correcting non-normal distributions can be useful for correctly detecting strength of effects (Abbott 2014). Our data showed moderate skewness (psychological need satisfaction: − .70; psychological need frustration: .38) and were therefore transformed to be normal. To evaluate heteroscedasticity in the model we examined cook’s distance (Fox 1991) and found that no contributors placed undue influence on the model.

Relations with all indicators are summarized and presented alongside effect sizes, represented in terms of partial correlations, in Table 2. Controlling for covariates (gender and latency; see Table 2 for their relations with dream themes and emotions), psychological need satisfaction did not relate to negative dream themes, *b* = − 1.95 (95% CI [− 5.45, 1.55]), wald = 1.18, *p* = .28, though individuals who experienced more psychological need frustration in their waking lives also reported more of these themes in their dreams, *b* = 3.37 (95% CI [0.55, 6.19]), wald = 5.48, *p* = .02.

**Table 2:** Results of regression analyses defining gender, dream latency, and need experiences (Study 1)

	Themes			Emotions					
				Negative			Positive		
	*b*	wald	*OR*	β	*t*	*pr*	β	*t*	*pr*
Gender	− 0.34	0.48	0.71	.13	1.86	.13	− .09	− 1.22	− .09
Latency	− 0.03	0.02	1.03	.05	0.77	.06	− .21	− 3.05**	− .22
Need satisfaction	− 1.95	1.18	29.08	.00	0.04	.03	.13	1.88	.14
Need frustration	3.37	5.48*	0.14	.32	4.64**	.32	− .03	− 0.47	.04

Significance tests are based on degrees of freedom of 192

*OR* odds ratio, *Pr* stands for partial regression coefficient

*p < .05, **p < .01

#### Dream emotions

A second set of analyses was conducted with Ordinary Least Squares (OLS) regression given that dream emotions were continuously distributed, but was otherwise structured similarly to our investigation of dream themes. See Table 2 for relations between dream emotions and both covariates and predictors, along with effect sizes for these. Contrary to our expectations, no relation was found between psychological need satisfying experiences and positive dream emotions, *b* = 1.04 (95% CI [− 0.05, 2.13]), *t*(192) = 1.88, *p* = .06, and psychological need satisfaction was also unrelated to negative dream emotions, *b* = − .02 (95% CI [− 1.01, 1.05]), *t*(192) = 0.04, *p* = .97. On the other hand, as expected, psychological need frustration was linked positively to negative dream emotions, *b* = 2.15 (95% CI [1.24, 3.06]), *t*(192) = 4.64, *p* < .001, but no relation was found with positive emotions: *b* = − .23 (95% CI [− 1.20, 0.70]), *t*(192) = − 0.47, *p* = .64.^1^

### Brief discussion

Study 1 findings indicated that individuals who experienced more psychological need frustration in their life in general reported having recurring dreams with more negative emotions, and which had somewhat more negative themes present in them. On the other hand, psychological need satisfaction did not relate to any dream experiences, not even in the case of positive emotions in dreams as we had hypothesized. Thus, it seems that psychological need experiences—and in particular need frustrating ones—may be more relevant for explicitly negative dream themes and emotions and relatively independent of positive dream emotions.

## Study 2

Study 2 aimed to replicate and extend the findings from Study 1 in two significant ways. First, we assessed psychological need experiences and dreams across three days, which allowed us to separate the within-person from the between-person variability in needs and dreams. The differentiation between these two levels provided the opportunity to examine whether the observed relation between psychological need frustration and dream content and emotions also applied at the day-to-day level. For themes reflective of psychological need frustration to surface in dreams, people need to experience an accumulation of psychological need frustration over longer periods of time. Thus, the question is whether variation in need-based functioning from day-to-day within a given person is sufficient for such experiences to play out in dreams. Second, we controlled for daily mood to account for the possibility that affectivity was driving the observed effects of need-based experiences, given that emotions have also been shown to relate to dream themes in previous research (Brown and Donderi 1986; Kallmeyer and Chang 1998). Technically, the observed dream effect of need-based experiences in Study 1 may be spurious, that is, driven by a third variable (i.e., mood) which we now control for. Congruent with Study 1, we again hypothesized a dual pathway model, with daily psychological need frustration relating to daily negative themes and negative dream emotions and daily psychological need satisfaction relating to daily positive dream emotions. These hypotheses were equally examined at the between-subject level, focusing on how more enduring need-related experiences link to dreaming across time.

### Method

#### Participants and procedure

Participants were 110 students and adults (79 women) at the University of Essex, with ages ranging from 18 to 61 years (*M* = 25.09, *SD* = 12.18). Participants were recruited for a study titled “three days in your life…” and took part in exchange for course credit or monetary compensation, based on their preference. We conducted the study throughout the course of a semester and data collection ended at the end of the academic year. Participants first completed an initial survey assessing person-level variables (i.e., general psychological need satisfaction and frustration), and then completed surveys on three evenings and the three mornings following, on days Monday to Thursday. We did not test participants on weekends to avoid measuring on days with irregular sleep patterns (Lund et al. 2010). Email reminders were sent at 7:00, 9:00, and 11:00 pm to all participants who had not completed the study by that time; responses were completed between 5:37 pm and 4:15 am (*M* = 10:47 pm). All participants were asked to complete morning responses immediately upon waking and the morning survey closed at 12:00 pm. Seventy-one percent of participants completed all three evening measures; 18% completed two evening measures, and 11% completed only one evening measure. On each evening, participants reported on their psychological need satisfaction from that day and their positive or negative mood from that day (described below). In the mornings they reported on whether they had remembered their dreams (participants remembered their dreams 45.8% of the time^2^), and if they remembered their dreams, they also reported on the positive and negative dream emotions and themes as in Study 1 (present study reliabilities were high: α = .83 and α = .90 for positive and negative emotions, respectively). Rates of missing responses are comparable to those found in previous research using dream diaries (Schredl and Hofman 2003).

### Additional materials

#### Person-level measures

##### General psychological need satisfaction and frustration

All participants completed the BPNSNFS (Chen et al. 2015) prior to beginning the diary study. Using the same scale and items as described in Study 1, two composite scores were created to reflect psychological need satisfaction, α = .79, and frustration, α = .81.

#### Day-level evening measures

##### Daily psychological need satisfaction and frustration

Participants reported on their daily psychological need satisfaction and frustration using the shortened version of the BPNSNFS (Chen et al. 2015; Van der Kaap-Deeder et al. 2017) from Study 1. Twelve items assessed the frustration and satisfaction of the basic psychological needs experienced that day using a 1 (*not at all true*) to 5 (*extremely true*) scale. Items included: “Today, I felt that my decisions reflected what I really want” (autonomy satisfaction) and, “Today, I felt that people who are important to me were cold and distant towards me” (relatedness frustration). Reliability for daily psychological need satisfaction averaged across days was α = .83 and α = .76 for daily psychological need frustration.

##### Daily mood

Participants responded to twenty items of the Positive and Negative Affect Schedule (Watson et al. 1988), assessing the degree to which they experienced negative (upset, ashamed) and positive (happy, hopeful) emotions. All items were rated on a scale of 1 (*not at all*) to 7 (*extremely*). Items were averaged to create a daily positive mood (α across all 3 days = .91) and a daily negative mood (α across all 3 days = .83) indicator.

### Results

#### Statistical controls

Preliminary correlations (Table 3) showed relations between gender and more negative dream emotions, in line with Study 1 findings. As a result and to be consistent with the previous study, Study 2 analyses controlled for gender.

**Table 3:** Descriptive statistics and correlations among measured variables (Study 2)

		1	2	3	4	5	6	7	8	9
Person-level measures										
1. Gender										
2. General need satisfaction	0.51 (.11)	− .10								
3. General need frustration	0.23 (.15)	− .06	− .43**							
Day-level measures										
4. Day positive mood	0.42 (.15)	− .24**	.67**	− .30**						
5. Day negative mood	0.24 (.13)	− .04	− .14*	.55**	− .03					
6. Day need satisfaction	0.54 (.09)	− .06	.53**	− .27**	.40**	− .07				
7. Day need frustration	0.32 (.16)	.03	− .43**	.56**	− .30**	.44**	− .45**			
8. Positive dream emotion	2.35 (.70)	− .09	.14	.04	.14	.10	.17	− .01		
9. Negative dream emotion	2.21 (.88)	− .18*	.04	.23*	.06	.18	.00	.17	− .25**	
10. Dream themes	0.60 (.49)	− .16	.05	.11	.09	.07	.01	.12	.00	.46**

*p < .05, **p < .01. Correlations presented at the day level: *n* = 260 for mood and need satisfaction; *n* = 119 when correlation involves dream emotion or content. Day-level measures were aggregated across the three days

#### Analytic strategy

Given the nested nature of the data, that is, days were nested within participants, hierarchical linear modeling (Bryk and Raudenbush 1992) was used which regressed dream outcomes from waking experiences defined both at the between- and within-subject level. To do this, we used HLM 7.01 (Science Software International [SSI]). HLM assumes data defined at level 1 to be normally distributed, but our level 1 predictors were moderately skewed (skewness ranged from .13 to .81); For consistency all predictors defined in these models were transformed to be normal. HLM is also better equipped to handle missing or unbalanced data than ordinary least squares (OLS) regression analyses because it uses full information maximum likelihood (ML) estimation which includes the missing data points in the analysis, and because it accounts for the random effects at Level 2 (Enders 2011; Enders and Peugh 2004; Little and Rubin 1989).

Specifically, models defined gender, general psychological need satisfaction and frustration at Level 2 (i.e., between-subject level), and daily positive mood, daily negative mood, daily psychological need satisfaction and frustration at Level 1 (i.e., within-subject level). Unconditional analyses conducted to attain the intraclass correlation (ICC) indicated that 23% of the variability in negative themes, 28% of the variability in positive dream emotions, and 40% of the variability in negative dream emotions, was between-subjects; in other words, much of the variability occurred on the day-to-day level. In line with recommendations (Bryk and Raudenbush 1992), variables at Level 1 were group centered whereas variables at Level 2 were grand centered. Given HLM accommodates and estimates missing data, all participant days were included in analyses regardless of dream recall on that day.

### Primary results

#### Dream themes

A model predicting negative themes controlled for gender at level 2 and both positive and negative waking-life affect at level 1. See Table 4 for relations with these constructs and effect sizes, represented in terms of Pearson coefficient, *r*, for all indicators tested. Accounting for control variables, there were no links between the psychological need experiences of individuals, in general (i.e., at Level 2), and daily dream themes, *ts* < 1.63, *ps* > .11. Furthermore, there were no links between day level (i.e., Level 1) psychological need experiences and daily dream themes, *ts* < 0.54, *ps* > .59.^3^

**Table 4:** results of hierarchical linear models including all variables defined at level 1 (day-level) and level 2 (person-level) (Study 2)

	Themes			Emotions					
				Positive			Negative		
	*b*	*t*	*OR*	*b*	*t*	*r*	*b*	*t*	*r*
Day-level measures									
Need satisfaction	2.26	0.54	9.61	2.81	2.45*	.47	-0.58	-0.28	− .05
Need frustration	1.66	0.51	5.23	− 1.83	− 2.14*	− .41	2.72	2.40**	.46
Positive affect	5.95	1.90	382.72	− 1.55	− 1.76	− .33	0.96	0.72	.14
Negative affect	5.42	0.24	225.47	0.89	0.92	.18	− 1.55	− 1.04	− .20
Person-level measures									
Gender	− .71	− 1.73	4.93	− .13	− 0.93	− .18	− 0.36	− 2.06*	− .39
Need satisfaction	2.88	0.57	17.89	1.81	2.16*	.41	1.72	1.90	.36
Need frustration	2.50	1.53	12.19	0.68	1.21	.23	1.33	2.12*	.40

Significance tests are based on degrees of freedom of 60 for person-level measures and 111 for day-level measures

*p < .05, **p < .01

#### Dream emotions

Models predicting both positive and negative dream emotions controlled for gender at Level 2 and both positive and negative waking-life affect at Level 1. At the individual-difference level, psychological need satisfaction linked positively to both positive emotions reported in dreams across nights, *b* = 1.91 (95% CI [0.21, 3.62]), *t*(111) = 2.45, *p* = .02. and positive emotions in one’s dreams on a given night, *b* = 2.81 (95% CI [0.56, 5.06]), *t*(111) = 2.45, *p* = .02. Conversely, daily psychological need frustration related to less positive dream emotions, *b* = −1.83 (95% CI [− 3.50, 0.16]), *t*(111) = − 2.14, *p* = .03.

Furthermore, individuals who were generally more need frustrated reported more negative emotions in dreams across days, *b* = 1.33 (95% CI [0.10, 2.56]), *t*(111) = 2.12, *p* = .02 and also reported more negative dream emotions on a given day, *b* = 2.72 (95% CI [0.50, 4.94]), *t*(111) = 2.12, *p* = .04, as was hypothesized. In contrast, there was no relation between either general or daily psychological need satisfaction and negative emotions in dreams (See Table 4).^4^

### Brief discussion

Study 2 explored links between psychological need experiences and dream emotions and themes, but extended these findings to analyses at the daily level and across days. Findings indicated that on days in which individuals felt especially need frustrated, they experienced their dreams as having more negative emotions and fewer positive emotions. In addition, those who reported more psychological need frustration in their daily lives in general also reported that they remembered more negative emotions in their dreams, while those who reported more psychological need satisfaction in general recalled more positive emotions in their dreams, suggesting that more enduring waking experiences carry into dreams, and conceptually replicating Study 1. Moreover, these effects were in evidence after controlling for daily positive and negative affect, indicating that relations were more than an indicator of positive or negative tendencies in responding, or of the negative affect in waking life that might be elicited by such psychological need experiences (Reis et al. 2000). Different from Study 1, need-based experiences, either more enduring or on a day-to-day level, were unrelated to dream themes.

### General discussion

The present research examined the extent to which waking-life experiences of psychological need frustration and satisfaction are expressed in recurring (Study 1) and daily (Study 2) dreams. We tested and found general support for our broad expectation that waking psychological need experiences are reflected in people’s dreams. By testing both recurring dreams and day-to-day variations in dream experiences we were able to examine more stable or cumulative effects as well as short-term correlates of both stable and everyday psychological need experiences. By examining how waking experiences are reflected in dreams, we aimed to contribute to current knowledge regarding how psychological need experiences are processed and manifest at less conscious levels. In addition, by examining the independent effects of psychological need satisfaction and frustration, we aimed to gain further insight into the fundamental dynamic differences between both, as assumed within the dual pathway model (Bartholomew et al. 2011; Vansteenkiste and Ryan 2013).

#### Dream themes

We found mixed support for the notion that experiences of psychological need frustration would be reflected in dream themes. In Study 1, those higher in general psychological need frustration reported recurring dreams characterized by negative dream themes, such as ‘falling’, ‘failing’ or ‘being attacked’; yet, such findings could not be replicated in Study 2. We provide two explanations for these findings. First, *recurring* dreams may be more sensitive to distressing psychological experiences that must be processed by the individual. In line with this, researchers and theorists have argued that recurring dreams challenge individuals to process the most pressing problems in their lives (Weiss 1964), and may be thought to result from individuals’ failure to adapt to challenging experiences (Brown and Donderi 1986; Klein et al. 1971). As such, dream content may be more affected by enduring need-based experiences, such as those assessed in Study 1. Alternatively, it might be that a third variable explains this relation when looking at the broad individual difference level over time. For example, lower general psychological well-being has been linked to recurring dreams, as has depression and other psychological disturbances (Brown and Donderi 1986; Cartwright and Romanek 1978; Renik 1981), but this possibility should be evaluated in future research.

Notably, there were no links between psychological need satisfaction and dream themes, which may be due to the fact that all of the dream themes we studied were negative. It may be that psychological need satisfaction would be more closely linked with positive themes in dreams, although such links may be difficult to detect because positive themes in dreams are more rare and subtle than negative ones (Curci and Rimé 2008; Schredl and Doll 1998).

If future research supports the role that dream themes are more closely linked to psychological need frustrations, which are thought to elicit psychological threat and dysfunction more so than merely the absence of psychological need satisfaction (Vansteenkiste and Ryan 2013), this would support theorizing that dream elements may be expressions of negative experiences that were difficult to process in waking-life (Freud 1913/2010; Jung 1948/1974; Perls et al. 1973). Such findings are congruent with the proposed *function* of dreaming as an opportunity for psychological integration of waking experiences. Within SDT, such self-integration, or the organization of important materials into one’s self, is thought to support behavioral regulation and learning (Payne 1985; Deci and Ryan 1991). In line with this view, it has been argued that dreams function to help integrate threatening or painful experiences into the self, and by doing so to gain mastery over waking life themes by replaying meaningful problems or conflicts in dream themes (Adler 1927/1963; De Monchaux 1993; Erikson 1954; Jones 1962; Jung 1948/1974). If Study 1 findings are replicated with recurring dreams, this would point to a functionality of dreams as helping to process psychological need frustrating experiences that are more threatening to the waking self. Alternatively, it may also be that those who are more capable of integrating psychological need frustration themes in their waking lives, for example, those who score higher on measures of emotional integration or self-congruence (Roth et al. 2014; Weinstein et al. 2012) may show weaker associations with dream contents given these have already been processed during waking hours.

It is important to recognize that while we focused on the subjective experience of psychological need frustration as a predictor of dream content in these studies, it might be the case that *need thwarting environments* are more responsible for shaping the contents of dreams. As an example, while it might be relatively easier to process one’s experience of being lonely (i.e., relatedness psychological need frustration), the *events* that brought on such feelings—in this case perhaps being socially rejected—might be more psychologically threatening and difficult to process in waking life. In this case as well, individuals might struggle to identify ways to cope with such events, and might be challenged by their broader implications for the self. In the current studies we did not assess the need thwarting context and, hence, cannot disentangle the experience of psychological need frustration as such from the events that gave rise to the experience. In the future, researchers might include measures of both need-relevant life events as well as experiences of need fulfillment or frustration to examine whether they differentially predict outcomes.

#### Emotional experiences

More robust than the association with dream themes, results supported the role of psychological need frustration in negative emotions in dreams, both when examining individual differences in (Study 1), and daily variations of (Study 2), being need frustrated. Psychological need satisfaction, on the other hand, did not relate to negative dream emotions in either study. Overall, the results indicated that individuals whose needs were frustrated reported more negative emotions in their dreams. To account for the possibility that these individuals were simply suffering from a general “cloud” of negative affect which colored their interpretation of real-life psychological need experiences and dream themes, in Study 2 we controlled for affect so as to account for the possibility that dreams would be influenced by emotions rather than actual psychological need experiences. Notably, in contrast to previous research which found a link between emotions and dreams (e.g., Brown and Donderi 1986; Kallmeyer and Chang 1998), in Study 2 affect did not relate directly to either dream themes or emotions, suggesting that dream emotions may be more robustly linked to need-laden experiences than recent emotions. We may understand this within the same theoretical framework as when interpreting the role of themes in dreams: Negative dream emotions may have directly resulted from distressing dream events, which as we note above, might represent the psyche’s attempt to process and make sense of particularly psychologically challenging waking experiences (Freud 1913/2010; Jung 1948/1974; Perls et al. 1973).

Further, reports of emotions in dreams may be more subject to the dreamer’s interpretation than reports of themes, yet this subjectivity may be of practical importance in its own right. Indeed, in Gestalt therapy such interpretations of dreams are thought to provide an important foundation for self-exploration and a basis for discussing more difficult, painful, or ambiguous aspects of one’s life; as such these interpretations offer opportunities for deeper self-insight and useful tools in therapy (Perls et al. 1973; Simkin 1972). It may be then that therapists can explore dream interpretations as a way of understanding important psychological need experiences, particularly with clients that are more easily threatened by more direct explorations (Newman 2002; Speisman 1959).

On the other hand, while psychological need satisfaction did not link to negative emotions in dreams in either study, daily variations in psychological need satisfaction related to more positive emotions in the subsequent night’s dreams. This finding was not found in Study 1 when examining links between psychological need satisfaction and positive emotions in recurring dreams, but this is not entirely surprising given our hypothesized associations here are less well-grounded in theory (e.g., Jung 1948/1974; Perls et al. 1973) than the hypothesized associations for psychological need frustration.

Overall, the finding that psychological need satisfaction was associated with more positive dream emotions in Study 2, and psychological need frustration with more negative dream themes and emotions is in line with the proposed dual pathway model within SDT which holds that psychological need frustration should be related more strongly to negative outcomes and psychological need satisfaction more strongly to positive outcomes (e.g., Bartholomew et al. 2011). The present research adds to this body of work by providing further evidence for the independent role of psychological need satisfaction and frustration at both the interpersonal and intrapersonal level, with these two constructs being related to differential outcomes (Vansteenkiste and Ryan 2013). In relating to dreams, the measure of psychological need frustration was a more informative predictor than a composite score of psychological needs would have been, and testifies to the incremental value of this approach.

#### Implications

The present findings also have implications for past work which has identified a relation between individual differences in materialism and mindfulness and dream themes and emotions (e.g., Kasser and Kasser 2001; Simor et al. 2011). Indeed, both materialism and mindfulness have been previously linked to lower and higher psychological need satisfaction, respectively (e.g., Brown and Ryan 2003; Dittmar et al. 2014). While materialism is thought to undermine psychological needs through the pursuit of extrinsic goals such as wealth and material successes which are inherently unsatisfying, it is proposed that mindfulness, which involves an open awareness of and receptivity to present experiences, relates to higher psychological need satisfaction through greater attunement to cues for and selection of psychological need satisfying activities (see Campbell et al. 2015). The results of the present research, in conjunction with past findings, suggest that psychological need experiences may be underlying mechanisms which help to explain why mindfulness and materialism relate to dream outcomes, an issue which could be explored in future research.

In addition, although past work has sought to highlight the differences between daily dreams and recurrent dreams (e.g. Brown and Donderi 1986), the results of the present research suggest that the pathway to both may be similar, particularly when dream themes are negative. While it is notable that recurring dreams present the same thematic elements over intervals of weeks, months or even longer, while daily dreams typically present more variation in thematic themes, the results of the present research suggest that psychological need frustration may play a central role in the etiology of both types of bad dreams. It seems plausible that when psychological need frustration persists over time, the unresolved psychological conflict may promote daily bad dreams to reoccur and develop into recurrent dreams.

### Limitations and suggestions for future research

The present research presents some notable limitations which should be taken into consideration when interpreting the results. Three of these have to do with the surveys used, and can be rectified in future research. First, all measures were retrospective in nature and therefore subject to recall bias. One way of reducing this bias would be to assess dream reports throughout the night in a sleep laboratory, directly after dreams are experienced. For example, participants’ could be woken at regular intervals and asked to record their dream into a tape recorder (e.g., McNamara et al. 2014). Notably, negative dream emotions and themes may also help to explain why psychological need frustration is predictive of poorer sleep (Campbell et al. 2017). A further concern, Study 1 asked participants to think back as far as they choose to their recurring dreams, and measured psychological need satisfaction ‘in general’, a potentially problematic method which may have resulted in participants reporting on psychological need satisfaction experienced more recently than their dream content; future research can use retrospective data which more carefully specifies and, hence matches, time periods for waking and sleep experiences. Third, to reduce participant burden during repeated measurements, we used a brief version of the BPNSNFS to measure psychological needs, and as such conclusions were drawn from a somewhat exploratory assessment of this construct. Although this brief version was used in past research (Van der Kaap-Deeder et al. 2017), in future work researchers could use the full scale for greater confidence in the findings.

In addition to these concerns, Study 2 showed large amounts (45.8%) of missing data, primarily due to participants not recalling their dreams, and results based on this study may have been affected by this. Although as previously noted, in research utilizing dream diaries people often fail to recall their dreams (e.g., Schredl and Hofman 2003). Thus, this is perhaps an issue inherent to the study of the daily dynamics in dreams. Furthermore, estimating missing values has previously been shown to be a reliable approach, even for large amounts of missing data (Enders 2011; Enders and Peugh 2004; Little and Rubin 1989) and our analyses of the truncated sample of participants who remembered their dreams produced similar results.

Third, the present research relied solely on self-reports of dreams and psychological need experiences. To address the issue of shared method variance, future studies could use more concrete and varied measurements of psychological need frustration. For example, implicit measures could be used to assess feelings of psychological need frustration or an experimental design could be used to examine the role of psychological need frustrating themes (e.g. failing a test or receiving negative feedback) on subsequent dreams at night. In this context, Van der Kaap-Deeder et al. (2016) demonstrated that experimentally induced negative feedback caused lasting rumination and lack of acceptance among self-critical individuals, a need-thwarting experience that may also manifest via dreams.

Furthermore, both studies were conducted with samples which were mainly comprised of students and therefore not representative of the general population. Hence, there is a need to replicate these results in more diverse community samples and across distinct cultures. Finally, an avenue for future work is the exploration of the salience (i.e., recall and vividness) of psychological need experiences as reflected in dream themes and emotions. Some early psychological approaches to dream recall have argued that when dreams are overly threatening, individuals are less likely to recall them (Lachman et al. 1962); whereas others have argued that overwhelming themes in dreams can increase recall because individuals become fixated on it (Wallach 1963); future studies can examine such questions in the context of psychological need experiences.

## Conclusion

In sum, to our knowledge the present research was the first to explore whether waking-life experiences of psychological need frustration and satisfaction are reflected in recurring and daily dreams. Both studies indicated that psychological need frustration may play a central role in eliciting negative dream themes and further suggested that psychological need experiences may especially relate to dream emotions, with psychological need frustration in particular being associated with negative dream emotions. Overall, these studies suggest that waking-life psychological need experiences are indeed reflected in our dreams.

## Footnotes

1 In our model predicting negative emotions in dreams, one participant showed undue influence but a second analysis removing this participant did not show a difference in the direction or significance of the effect.

2 The number of days in which dreams were remembered did not correlate with either psychological need satisfaction, *r* = − .06, *p* = .55, or psychological need frustration, *r* = − .09, *p* = .37; similarly, the number of days participants completed surveys did not correlate with either psychological need satisfaction, *r* = − .11, *p* = .27, or psychological need frustration, *r* = − .11, *p* = .29.

3 Additional analyses conducted with recalled dream days only showed effects similar in direction and significance.

4 Analyses showed no heterogeneity in the data,* χ* ^2^s < 6.0,* ps* > .50.
